# PAUF as a Target for Treatment of High PAUF-Expressing Ovarian Cancer

**DOI:** 10.3389/fphar.2022.890614

**Published:** 2022-05-06

**Authors:** Yeon Jeong Kim, Fen Jiang, Jin Park, Hyeon Hee Jeong, Ji Eun Baek, Seung-Mo Hong, Seong-Yun Jeong, Sang Seok Koh

**Affiliations:** ^1^ Department of Biomedical Sciences, Dong-A University, Busan, South Korea; ^2^ Innovative Discovery Center, Prestige Biopharma, Busan, South Korea; ^3^ Department of Pharmacology, Inje University College of Medicine, Busan, South Korea; ^4^ Asan Institute for Life Sciences, Asan Medical Center, University of Ulsan College of Medicine, Seoul, South Korea; ^5^ Department of Pathology, Asan Medical Center, University of Ulsan College of Medicine, Seoul, South Korea

**Keywords:** PAUF, ovarian cancer, molecular targeted therapy, monoclonal antibody, combination chemotherapy

## Abstract

Pancreatic adenocarcinoma up-regulated factor (PAUF) plays an important role in tumor growth, metastasis, and immune evasion in the pancreatic tumor microenvironment, and recent studies suggest an association between PAUF expression and poor prognosis in ovarian cancer patients. The current study aimed 1) to characterize the potential tumor-promoting role of PAUF in ovarian cancer, using *in vitro* and *in vivo* models, including a PAUF-knockout OVCAR-5 cell line, and 2) to explore the potential therapeutic effects of an anti-PAUF antibody for ovarian cancer. Recombinant PAUF significantly increased tumor metastatic capacity (migration, invasion, and adhesion) in all the ovarian cancer cell lines tested, except for the OVCAR-5 cell line which expresses PAUF at a much higher level than the other cells. PAUF-knockout in the OVCAR-5 cell line led to apparently delayed tumor growth *in vitro* and *in vivo*. Furthermore, the administration of an anti-PAUF antibody exhibited notable sensitizing and synchronizing effects on docetaxel in mice bearing the OVCAR-5 xenograft tumors. Taken together, this study shows that the expression level of PAUF is an independent factor determining malignant behaviors of ovarian cancer and, for the first time, it suggests that PAUF may be a promising therapeutic target for high PAUF-expressing ovarian cancer.

## Introduction

Ovarian cancer is the deadliest gynecological cancer, as most of the patients are diagnosed at advanced stages, where less than one third of the patients can survive more than 5 years after the first diagnosis (SEER*Explorer, United States, National Cancer Institute). Up to date, continuing efforts are made in searching for common diagnostic biomarkers and therapeutic targets for pathologically and clinically distinct cancer types. Attempts of targeting the trending tumor associated antigens PD-1 and PD-L1, have achieved great success in certain cancers, but made limited progress in other cancer settings including ovarian (Zhu et al., 2021) and pancreatic (Feng et al., 2017). Other new targeted treatments including VEGF inhibitors and PARP inhibitors received regulatory approvals, but surgery and platinum-based chemotherapies are still the current standard of care for ovarian cancer (Beachler et al., 2020). Achieving early diagnosis as well as improving the efficacy and reducing the resistance of the chemotherapies have remained clinically relevant but challenging in ovarian cancer treatment.

Previously, we have discovered a secretory protein that is expressed at a much higher level in pancreatic ductal adenocarcinoma (PDAC) than in non-cancer pancreatic tissue and named it pancreatic adenocarcinoma up-regulated factor (PAUF) (Kim et al., 2009). Since then, there has been a continuing interest in establishing the biological functions of PAUF, and it is now clear that PAUF is a tumor-promoting molecule and exerts its cellular functions through both autocrine and paracrine mechanisms. For example, the autocrine signals activate pathways in pancreatic cancer cells such as CXCR4 axis (Lee et al., 2010; Park et al., 2011), AKT/beta-catenin (Cho et al., 2011; Cho et al., 2012), FAK (Lee et al., 2011) to promote tumor growth, metastasis, and resistance to chemotherapies, and the paracrine signals are delivered to other cells such as endothelial cells to induce tumor angiogenesis (Kim et al., 2013), and to immune cells such as myeloid-derived suppressor cells (MDSCs) to induce tumor evasion and tumor suppression (Song et al., 2016).

In recent years, studies have found that PAUF also expresses in ovarian cancer samples at varied levels (Kim S. J et al., 2014; Choi et al., 2018), and patients with elevated PAUF expression, compared to those with low PAUF expression, were associated with less favorable treatment outcomes, such as chemotherapy resistance, around 2-fold higher risk of disease progression and death (Kim S. J et al., 2014; Choi et al., 2018).

However, the role of PAUF in ovarian cancer development has not been clearly understood at the molecular level, and whether PAUF can be used as a therapeutic target of ovarian cancer awaits more scientific evidence. This exploratory study aimed 1) to characterize the functions of PAUF in ovarian cancer using cell-based assays and mouse xenograft experiments and 2) to further evaluate whether an anti-PAUF antibody can become a potential ovarian cancer treatment.

## Materials and Methods

### Cell Culture and Reagents

Human ovarian cancer cell lines (HeyA8, SK-OV-3, OVCAR-5, OVCAR-8) were obtained from the National Cancer Center of Korea. The Lenti-X^TM^ 293T cell line was obtained from Clontech (Mountain View, CA, United States). Cells were maintained at 37°C in a humidified atmosphere containing 5% CO_2_ and cultured in the medium recommended by the suppliers: Roswell Park Memorial Institute (RPMI) 1640 for HeyA8, SK-OV-3, OVCAR-5, and OVCAR-8 and Dulbecco’s modified Eagle’s medium (DMEM) for Lenti-X^TM^ 293T. Culture media were supplemented with 10% fetal bovine serum (FBS), 2 mM L-glutamine, 1 mM sodium pyruvate, 100 μg/ml streptomycin, and 100 IU/ml penicillin. RPMI 1640, DMEM, and FBS were purchased from GE Healthcare Life Science. Cells were periodically observed by monitoring cell morphology and growth rates, and *mycoplasma* contamination was monitored using a *mycoplasma* detection kit (Lonza, Basel, Swiss). Authentication of the cell lines was done using short tandem repeat (STR) profiling by the National Cancer Center of Korea with proper STR references. Recombinant PAUF (rPAUF) protein was prepared as previously described (Lee et al., 2010). Humanized anti-PAUF monoclonal antibody was prepared as described in the Patent Cooperation Treaty (PCT) WO2019022281A1. Human IgG isotype control (Cat. no. 31154, Thermo Fisher Scientific, Waltham, MA, United States) was used as a control for IgG. Antibodies against ERK (Cat. No. 9102S), p-ERK (Cat. No. 9101S), Src (Cat. No. 2108S), p-Src (Cat. No. 2105S), AKT (Cat. No. 4691S), p-AKT (Cat. No. 13038S) and Cas9 (Cat. No. 14697) were obtained from Cell Signaling Technology (Danvers, MA, United States). Anti-β-actin antibody was obtained from Santa Cruz Biotechnology (Dallas, TX, United States). The densities of the blots were quantified using the AzureSpot software version 14.0 (Azure Biosystems, Sierra, CA, United States).

### Sandwich ELISA

For measurement of secreted PAUF, cells were seeded at 7 × 10^5^ cells per well and cultured for 3 days to reach 80% confluency, when most cells entered the Log Growth Phase and were actively proliferating. The culture supernatant was then concentrated using a Vivaspin column (Cat. No. VS.0601, Sartorius, Gottingen, Germany) per manufacturer’s recommendation. Detection of PAUF by ELISA was as described previously (Choi et al., 2018).

### Generation of Pancreatic Adenocarcinoma Up-Regulated Factor-Knockout Ovarian Cancer Cell Line

A PAUF-knockout OVCAR-5 cell line (thereinafter PAUF K/O) was generated using Lenti-X^TM^ CRISPR/Cas9 genome editing. For sgRNA, we used the CRISPR/Cas9 design tool (http://crispr.mit.edu) to select key regions of the genome for protein function and to search for sgRNA (Shalem et al., 2014). Target specificity of the sgRNA sequence to PAUF was verified by using the BLAST search in NCBI. The oligonucleotides of the sgRNA sequence (5′-CAC​CGG​ACT​ACG​ACC​ATG​AAA​TCA​C-3′ and 5′-AAA​CGT​GAT​TTC​ATG​GTC​GTA​GTC​C-3′), were annealed and inserted into the BsmBI (Enzynomics) site of LentiCRISPR vector (Addgene, Watertown, MA, United States). The mock control sequence (5′-GTT​CCG​CGT​TAC​ATA​ACT​TA-3′) had no other genomic matches (Kearns et al., 2014). Confirmation of Cas9 transduction was performed by quantitative reverse transcription real-time polymerase chain reaction (RT-qPCR) and Western blot analysis of Cas9. The primer sequences for Cas9 RT-qPCR were 5′-GGA​CTC​CCG​GAT​GAA​CTA-3′ and 5′-TCG​CTT​CAG​CTT​AGG​TA-3’. The original OVCAR-5 cell line was designated as the wild-type (WT) control.

### Migration and Invasion Assays

Migration and invasion assays were performed in Transwell® plates purchased from Corning. To analyze cell invasion, the downside surface of the transwell insert membrane was coated with Matrigel^TM^ (BD Biosciences, Franklin Lakes, NJ, United States) while in cell migration assays it was not. The lower chambers were filled with standard medium. Cancer cells (HeyA8, SK-OV-3, OVCAR-8, OVCAR-5, or PAUF K/O, seeded at 9 × 10^3^ to 1 × 10^5^ cells/200 μl, depending on cell characteristics) were seeded into the upper chambers containing rPAUF (0.5 μg/ml, a concentration estimated to be sufficient to simulate maximum PAUF effects in all cell lines to be tested), anti-PAUF antibody (10 μg/ml, a concentration estimated to be sufficient to maximumly neutralize PAUF protein in all cell lines to be tested), or human IgG control (10 μg/ml) in serum-free culture medium. After incubation for 20 h at 37°C, the cells on the upper surface of the transwell insert were completely removed with a moist cotton swab. For both assays, the cells were fixed with methanol and stained, then counted and photographed by microscopy at ×100 magnification. All assays were performed on duplicate samples in three independent experiments. Data are presented as mean ± SEM of three independent experiments, and representative images are shown.

### Adhesion Assays

Adhesion assays were performed using a 24-well plate coated with collagen type I (Sigma-Aldrich, Saint Louis, MO, United States). Cancer cells (HeyA8, SK-OV-3, OVCAR-8, OVCAR-5, or PAUF K/O, 1–3 × 10^5^ cells/ml, depending on cell characteristics) were seeded in each well and unbound cells were removed by washing twice with PBS. Cell adhesion was quantified by counting the number of stained cells. All assays were performed on triplicate samples in three independent experiments. Data are presented as mean ± SEM of three independent experiments.

### Western Blot Assays

OVCAR-8 cells were seeded in a 100 mm culture dish at 7 × 10^5^ cells/10 ml and reached 70–80% confluency. The cells were then incubated in a serum-free medium for 15 h. After the serum starvation, the cells were treated with rPAUF (0.1 μg/ml) and incubated at 37°C for 0, 5, 10, 60, 120, and 240 min. Cells lysates were prepared in RIPA buffer (50 mM Tris-Cl, 150 mM NaCl, 1% sodium deoxycholate, 5 mM EDTA, 30 mM Na_2_HPO_4_, 50 mM NaF, 1 mM Na_3_VO_4_), and then were subjected to SDS-PAGE and transferred to a NC membrane (GE Healthcare, Chicago, United States). The membranes were probed with an appropriate primary antibody, and subsequently with a secondary horseradish peroxidase-conjugated antibody. The intensity of band was detected by Azure C300 gel imaging system (Azure Biosystems, CA, United States).

### Proliferation Assays

Proliferation assays were performed using alamarBlue (Invitrogen, Waltham, MA, United States) and WST-1 (Roche Diagnostics GmbH, Mannheim, Germany) assays. The alamarBlue assay was carried out according to manufacturer’s instructions. Briefly, cancer cells (Mock or PAUF K/O) were seeded at 1 × 10^3^ cells per well in 96-well plates and fluorescence was quantified using a microplate reader. The WST-1 assay was carried out according to manufacturer’s instructions. Briefly, cancer cells (Mock or PAUF K/O) were seeded at 3 × 10^3^ cells per well in 96-well plates and absorbance was quantified using a microplate reader. Three independent experiments were performed with duplicate samples in each. Data are presented as mean ± SEM of three independent experiments, and representative images are shown. *p < 0.05.

### Animal Experiments

The OVCAR-5 cell-derived xenograft tumor models were established in 6 week-old female BALB/c athymic nude mice (Japan SLC, Hamamatsu, Japan). For the tumorigenesis test, OVCAR-5 or its derivative cells were cultured and resuspended in saline, and 1 × 10^6^ cells were subcutaneously implanted into the right hind leg of the mice. For the drug treatment efficacy test, mice were implanted with the OVCAR-5 cells, and treatments were given after the average tumor volume reached 100 mm^3^. The mice were grouped into four groups (*n* = 15/group) based on the tumor volume. Human IgG control and anti-PAUF antibody were administered intravenously from day 0 at a dose of 10 mg/kg, twice a week (3–4 days of intervals) for 4 weeks. Docetaxel (Sanofi-Aventis) was administered intravenously at a single dose of 10 mg/kg on day 0. And docetaxel + anti-PAUF were given *via* the same regimens of the two drugs. Tumor volume (V) was calculated by measuring two perpendicular diameters with a caliper and using the formula of V= (a^2^×b)/2, where a and b are the shortest and longest diameters, respectively. Mice that reached the euthanasia criteria of tumor volume ≥2000 mm^3^ were euthanized by carbon dioxide (CO_2_) inhalation. On day 37, >90% mice died in all except the combination treatment groups, the study was then ended, and all the mice were sacrificed after the final measurement of tumor volume. All the survived animals on day 37 were counted as censored data. Body weight was measured twice a week (3–4 days of intervals), and survival was recorded. Tumor growth inhibition (TGI) rates (%) were calculated using the formula of TGI = 100-(V_T_/V_C_)*100, where V_T_ indicates mean tumor volume in treatment groups, and V_C_ indicates mean tumor volume in control group. All animal studies were performed according to the protocol (2019-12-176) approved by Institutional Animal Care and Use Committee of Asan Institute for Life Sciences, Korea.

### Statistical Analysis

For *in vitro* experiments, comparisons of migration, invasion, or adhesion between different treatments were analyzed using two-way ANOVA for multiple cell lines or Mann-Whitney test for single cell line, comparisons between multiple groups were analyzed using Kruskal–Wallis tests. Multiple comparisons within one experimental assay were corrected using the false discovery rate (FDR). For comparison of cancer cell proliferation or tumor growth over a period between control and PAUF K/O groups was analyzed using a linear mixed model. In the OVCAR-5 xenograft model, comparison of tumor volume between multiple treatment groups with human IgG control groups on different days was conducted using Dunnett’s multiple comparisons tests. Survival was analyzed by the Kaplan-Meier method, and comparisons between groups were analyzed by the Log-rank tests, *p* values were corrected using FDR. Results were considered significant when *p* < 0.05.

## Results

### Enhancement of Pancreatic Adenocarcinoma Up-Regulated Factor Expression Promoted Cancer Metastatic Capacity

**FIGURE 1:**
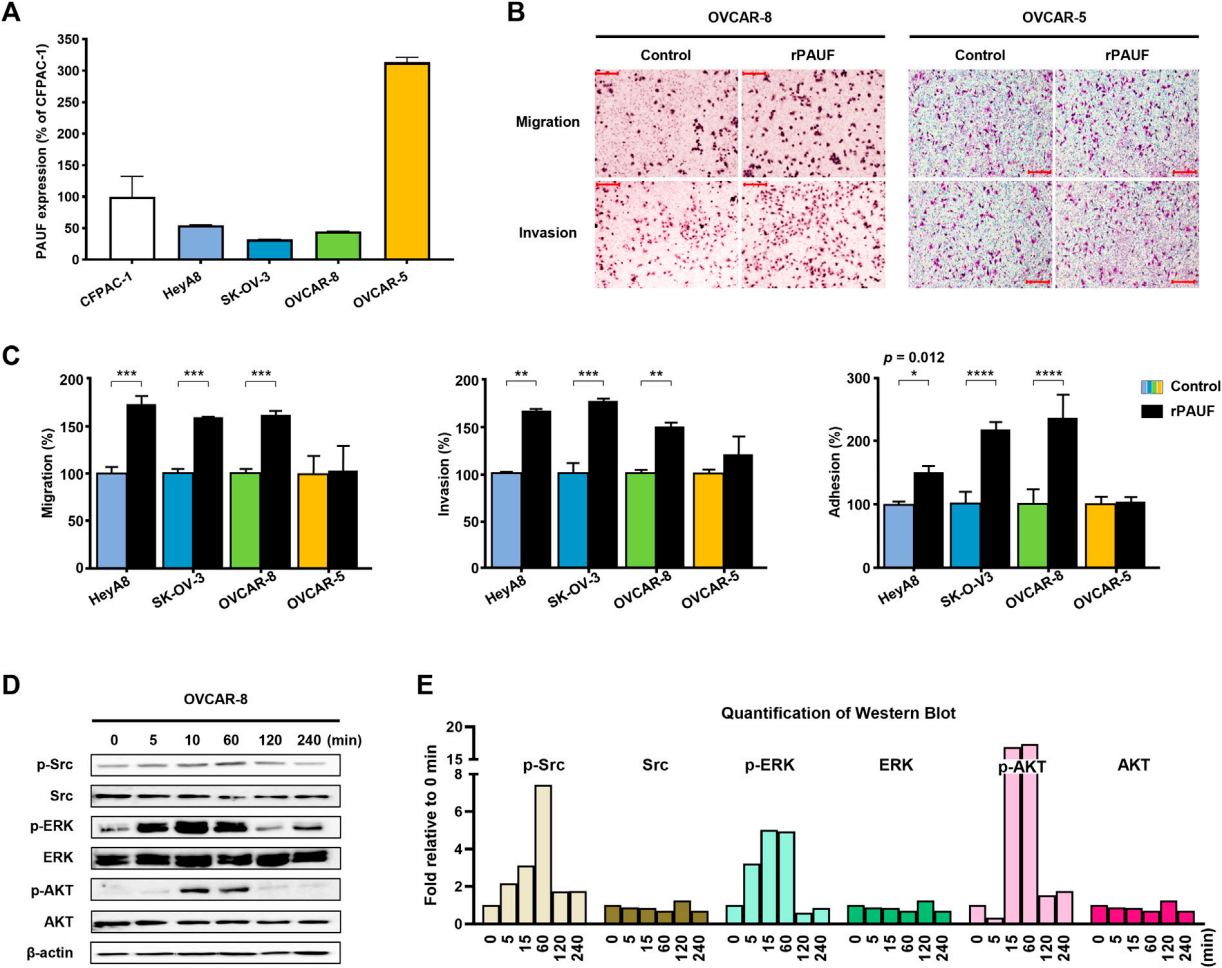
Enhancement of PAUF expression promoted cancer metastatic capacity in a baseline PAUF dependent manner. **(A)** Expression level of PAUF in ovarian cancer cells relative to pancreatic cancer cell CFPAC-1 was determined by Sandwich ELISA. **(B)** Effects of recombinant PAUF (rPAUF, 0.5 μg/ml) and PBS control on metastatic capacity (migration, invasion, and adhesion) of ovarian cancer cells. **(C)** Microscopy images (at ×100 magnification) of migration and invasion assay results in OVCAR-8 and OVCAR-5 ovarian cancer cells. Scale bar: 100 μm. **(D)** Western blot images and **(E)** image quantification showed time-dependent effects of rPAUF treatment (0.1 μg/ml) on Src, ERK, and AKT expression in OVCAR-8 cell be-tween 0 and 240 min. The original images of blots are shown in Supplementary Figure S1. ^∗^ *p* < 0.05, ^∗∗^ *p* < 0.01, ^∗∗∗^ *p*<0.001, ^∗∗∗∗^ *p* < 0.0001.

The levels of PAUF expression in the supernatants of human cancer cell culture (one pancreatic cancer cell CFPAC-1 and four ovarian cancer cells) were measured by sandwich ELISA assays (Figure 1A). Among the four ovarian cell lines tested, three cell lines showed lower PAUF expression than the CFPAC-1 cell, a cell line known to express relatively high-level of PAUF (Kim S. K et al., 2014), and the highest level of PAUF expression was discovered in the OVCAR-5 cells, which was around 3.1-fold higher than the CFPAC-1 cell, and 5.7-, 9.7-, and 7.0-fold higher than the HeyA8, SK-OV-3, and OVCAR-8 cell lines, respectively.

Next, the impact of rPAUF on the metastatic capacity of ovarian cancer cell lines was characterized in the four human ovarian cancer cell lines. As shown in Figure 1B and Figure 1C, the migration, invasion, and adhesion activities increased significantly (by 60–100%) by rPAUF treatment in all but the OVCAR-5 cells.

The activation of intracellular signaling pathway of the OVCAR-8, a cell with strong response to rPAUF treatment, showed involvement of Src, ERK, and AKT (Figure 1D), and the quantification of western blot is shown in Figure 1E.

### Knockout of Pancreatic Adenocarcinoma Up-Regulated Factor Caused Loss of Cancer Malignant Traits

**FIGURE 2:**
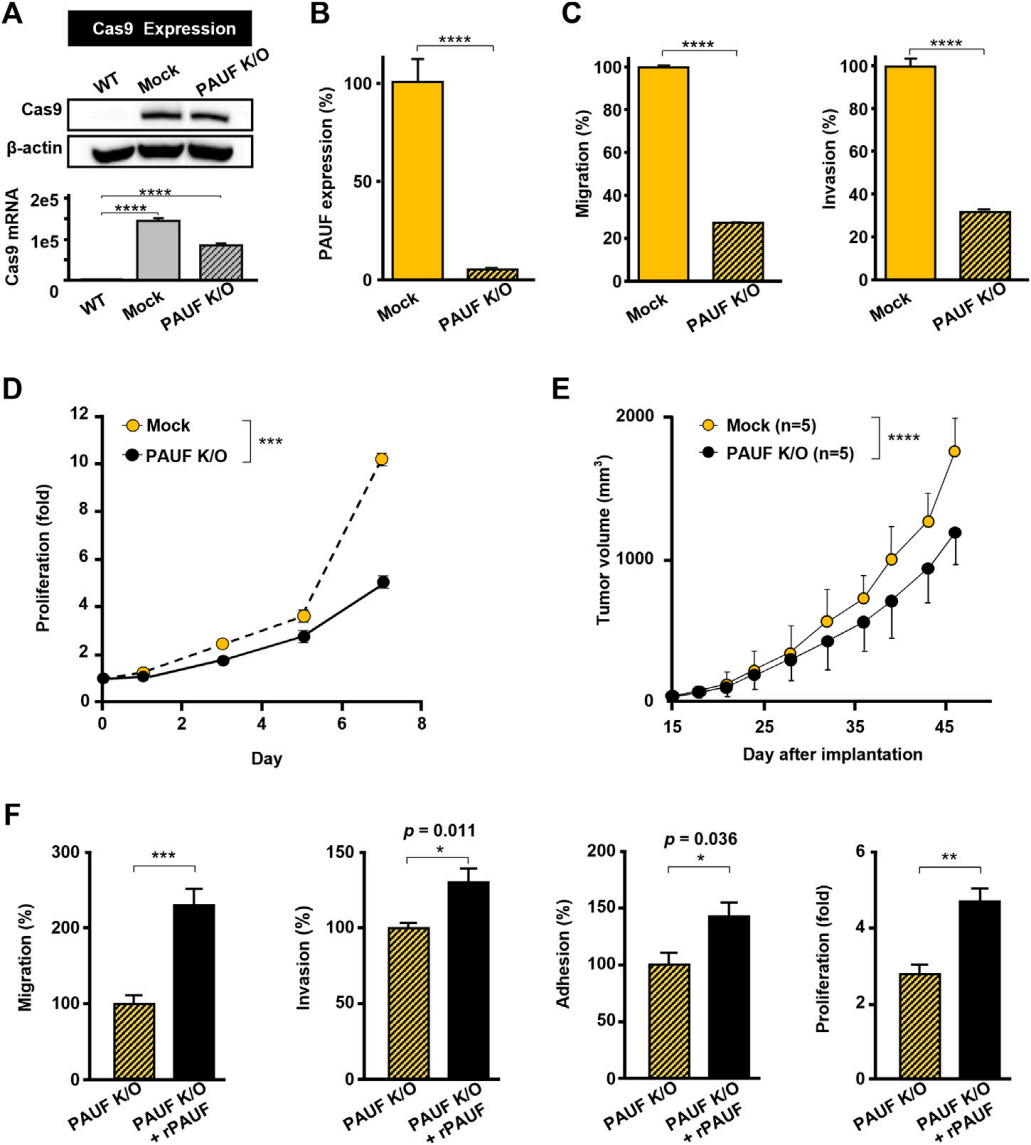
Knockout of PAUF caused loss of malignant traits in OVCAR-5 ovarian cancer cell, which was restored by recombinant PAUF (rPAUF) treatment. **(A)** Western blot (upper) and RT-PCR (lower) assays of Cas9 in OVCAR-5 wild-type (WT, without Cas9 transduction), Mock (only Cas9 transduction), and PAUF K/O (Cas9 transduction + PAUF gene deletion) cells to indicate a successful transduction of Cas9 protein. The original images of blots are shown in Supplementary Figure S2. **(B)** Sandwich ELISA of PAUF expression in OVCAR-5 Mock and PAUF K/O cells. **(C)** Migration and invasion of Mock and PAUF K/O cells. **(D)** Proliferation of OVCAR-5 Mock and PAUF K/O cancer cells *in vitro* were significantly different, analyzed using a linear mixed model. **(E)** Tumor growth in OVCAR-5 Mock and PAUF K/O xenograft mouse models were significantly different, analyzed using a linear mixed model. **(F)** Effects of rPAUF (0.5 μg/ml) on migration, invasion, adhesion, and proliferation of OVCAR-5 PAUF K/O cells. ^∗^ *p* < 0.05, ^∗∗^ *p* < 0.01, ^∗∗∗^ *p* < 0.001, ^∗∗∗∗^ *p* < 0.0001.

A PAUF-knockout OVCAR-5 cell line (PAUF K/O) was successfully generated, which was confirmed by immunoblot analysis and RT-qPCR (Figure 2A). The PAUF expression was reduced by over 90%, compared to the OVCAR-5 cell line (Figure 2B). Meanwhile, PAUF K/O showed significantly reduced metastatic capacity compared to the Mock cell line (migration and invasion reduced by 72% and 68%, respectively) (Figure 2C). PAUF K/O cells also proliferated at a slower rate than the OVCAR-5 cells (50% lower cell number on day 7) (Figure 2D). In BALB/c athymic nude mouse, PAUF K/O resulted in significantly delayed tumor growth. It took a mean of 32 days for the Mock tumors to reach 500 mm^3^ compared to 36 days in the PAUF K/O group. At the endpoint of the experiment on day 46, the average tumor volumes in the PAUF K/O group were much smaller [1,200 (range: 800-1,300) mm^3^] than in the Mock group [1,800 (range: 1,500-2,000) mm^3^], correlating to a tumor growth inhibition (TGI) of 33% from the PAUF-knockout (Figure 2E). In the PAUF K/O cells, supplement of rPAUF significantly reversed the loss of malignant traits (migration, invasion, adhesion, and proliferation) caused by the PAUF-knockout (Figure 2F).

### Anti-Pancreatic Adenocarcinoma Up-Regulated Factor Antibody Showed Anti-Tumor Effects in Human Ovarian Cancer Models

**FIGURE 3:**
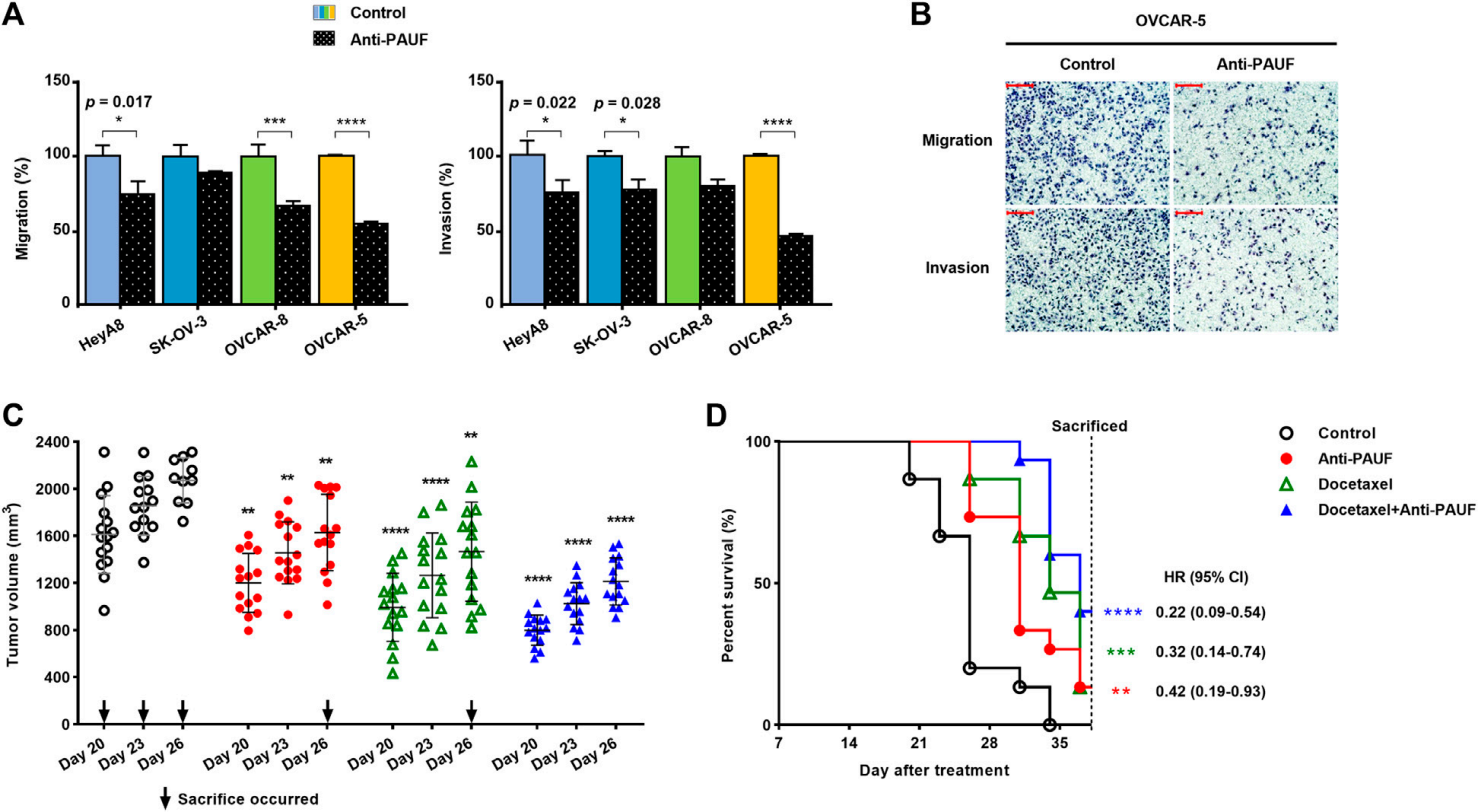
Anti-PAUF antibody demonstrated tumor-suppressing effects against ovarian cancers *in vitro* and *in vivo*. **(A)** Effects of anti-PAUF antibody (10 μg/ml) and human IgG control (10 μg/ml) treatment on migration and invasion of OVCAR-5 ovarian cancer cells *in vitro*. **(B)** Microscopy images (at ×100 magnification) of migration and invasion assay results in OVCAR-5 cells. Scale bar: 100 μm. **(C)** Individual tumor volume on day 20, 23, and 26 (the individual curves and mean value of tumor volume from the entire study period can be found in Supplementary Figure S3A and Supplementary Table S1 and **(D)** survival curves in OVCAR-5 xenograft mouse model, after receiving treatments (started from day 0) of human IgG control, anti-PAUF antibody (10 mg/kg, twice/week, ×4 weeks), docetaxel (10 mg/kg, once, on day 0), or docetaxel + anti-PAUF antibody (via the same regimens of the two drugs). Hazard ratio (HR) is the probability a treated mouse may die during the study period vs. untreated. ^∗^ *p* < 0.05, ^∗∗^ *p* < 0.01, ^∗∗∗^ *p* < 0.001, ^∗∗∗∗^ *p* < 0.0001.

The anti-PAUF antibody inhibited metastatic capacity (migration and invasion) in all the ovarian cancer cell lines tested (i.e., HeyA8, SK-OV-3, OVCAR-8, and OVCAR-5) (Figure 3A), but the inhibitory activity was the most significant in the OVCAR-5 cell line (Figure 3B).

For the anti-tumor treatment study in the human ovarian cancer xenograft mouse model, to better observe the trend in tumor volume changing and for simplicity, individual tumor volume (measured before sacrifice) data from the last half of the study (after day 17) are presented (Figure 3C). Between day 20 and day 26, all the three treatment groups (anti-PAUF, docetaxel, and docetaxel + anti-PAUF combination) demonstrated significantly reduced tumor growth, compared to the human IgG control group. After day 26, only three mice left in the control resulting in insufficient control data for analyses. A Kaplan-Meier analysis indicated that, compared to the human IgG control, all the three treatments significantly prolonged the animals’ survival, the greatest improvement was seen in the docetaxel + anti-PAUF combination group, followed by docetaxel group and anti-PAUF group (Figure 3D). The individual curves and mean value of tumor volume from the entire study period can be found in Supplementary Figure S3A and Supplementary Table S1. The TGI rates in the four groups were presented in Supplementary Figure S3B There was no difference in body weight among the test groups (Supplementary Figure S3C).

## Discussion

The current study provides lines of evidence that PAUF may represent a novel therapeutic target for ovarian cancer expressing a high-level of said molecule. First, manipulation of the expression level of PAUF, achieved through either exogenous enrichment (recombinant PAUF, rPAUF) or CRISPR-Cas9 knockout, led to notable changes in tumor metastatic capacity (i.e., migration, invasion, and adhesion) in human ovarian cancer cells. And rPAUF activated the same intracellular signaling pathways in ovarian cancer cells as those observed in pancreatic cancer cells. Second, treatment of a PAUF-neutralizing antibody led to suppressed malignant traits in the OVCAR-5, a high PAUF-expressing cancer cell, and remarkably sensitized and synchronized anti-tumor effects of the ovarian cancer chemotherapy docetaxel in mice bearing the OVCAR-5 xenograft tumors.

Recent cancer databases such as the Cancer Cell Line Encyclopedia (CCLE) have greatly facilitated research on cancer biomarkers. The RNA expression data of the ZG16B gene (the PAUF coding gene) in cancer cell lines (Supplementary Figure S4), show that high levels of PAUF expression can be found in a much higher portion of breast cancer and gastrointestinal cancer (pancreas, bile duct, gastric, and colorectal) cells, while ovarian cancers express a relatively low level of PAUF on average, but a small portion (around 10%) of the ovarian cancer cells expressed high level of PAUF. Our study shows that the OVCAR-5 cell line expresses PAUF at levels 5- to 10-fold higher than the other ovarian cancer cells tested, and at levels more than 3-fold higher than the CFPAC-1 pancreatic cancer cell. On the other hand, since PAUF expression varies largely among cancer subtypes, it is impossible to use a universal cut-off value to indicate high and low PAUF expression in different cancer types; a “low” PAUF expression level in breast cancer may be a “high” level in ovarian cancer, which may partly explain why the prognostic value of PAUF expression observed in breast cancer (Lu et al., 2021) is discrepant from other cancers.

In this study, artificial manipulation of the PAUF expression level in ovarian cancer cells resulted in notable changes in the cells’ malignant traits (i.e., tumor growth, metastasis), in a baseline PAUF expression-dependent manner. Increasing PAUF level using rPAUF largely promoted tumor migration, invasion, and adhesion in three out of four ovarian cancer cell lines tested. The PAUF-mediated intracellular signaling pathways activation observed in ovarian cancer cells were similar to that in pancreatic cancer cells (Lee et al., 2010; Lee et al., 2011), suggesting that PAUF may represent a common tumor-promoting factor between the two cancer types. Interestingly, rPAUF did not exert such tumor-promoting effects in the OVCAR-5 cell line, which expressed a high baseline level of PAUF (Figure 1A), however, when the PAUF gene was successfully knocked out from the OVCAR-5 cell line (Figure 2A and Figure 2B), not only the migration and invasion of the cancer cell were significantly reduced by about 70% (Figure 2C), the rPAUF-mediated migration/invasion/adhesion promoting effects were also much stronger (Figure 2F), manifesting a saturated tumor-promoting effect of PAUF in the OVCAR-5 cell line, as well as a dominant role of PAUF in defining this ovarian cancer cell line’s metastatic capacity.

It should be noted that the current *in vitro* study focused on metastasis promoting capacity of PAUF since PAUF-induced ovarian cancer proliferation and anti-PAUF mediated tumor growth suppression were reported previously (Choi et al., 2018). Therefore, the proliferation assay was only conducted in the PAUF K/O cancer cell but not conducted in other ovarian cancer cells. There is no natural inducer of PAUF identified so far, but in a genomic network-based study (Kim et al., 2018), a few genes, such as AGR2 and POM121, were discovered to be closely and positively correlated with PAUF expression in cervical cancer settings; finding out whether inducers for these genes (Zhang et al., 2005) also induce PAUF expression and identifying natural PAUF inducers will remain clinically meaningful tasks for cancer prevention and treatment, particularly for those ovarian cancers that express relatively low level of PAUF.

Based on the PAUF-knockout effects, we tested a newly engineered humanized anti-PAUF antibody for its tumor-suppressing activity against ovarian cancer. The biological characteristics of this protein were described in the PCT WO2019022281A1. In the *in vitro* experiments, the anti-PAUF antibody treatment resulted in suppressed cancer migration and invasion in all the ovarian cancer cells tested, with the highest suppression observed in the OVCAR-5 cell line, as expected. In the OVCAR-5 xenograft mouse model, the anti-PAUF antibody moderately but significantly slowed down ovarian tumor growth, and docetaxel attained a higher tumor-suppressing effect in average than in the anti-PAUF group. However, docetaxel demonstrated much wider inter-individual variation in the anti-tumor efficacy. Contrastingly, the combination of docetaxel and anti-PAUF achieved not only the highest, but also the most homogenous anti-tumor effect among all the treatment groups. This result confirmed the role of anti-PAUF treatments as a sensitizer and synchronizer for cytotoxic treatments, which were suggested in previous *in vitro* studies, where targeting PAUF downregulated drug efflux transporters on the surface of cancer cells (Gao et al., 2016; Cho et al., 2017) and induced cell cycle arrest (Liu et al., 2013; Escudero-Paniagua et al., 2020). Meanwhile, the combination group having the most potent anti-tumor efficacy was also supported by a nearly 5-fold improved survival in this group (HR = 0.22, HR is the probability a treated mouse may die during the study period vs. untreated), compared to the control group. Our animal study, along with the *in vitro* studies, formed the rationales underlying the combination of anti-PAUF and chemotherapies for cancer treatment.

Multiple strategies have been developed to target PAUF, such as engineering a RNA aptamer that can specifically binds to PAUF (Kim et al., 2011) or a trans-splicing ribozyme that was armed with suicidal mechanism (Kim Y. H et al., 2014), or directly targeting PAUF using an anti-PAUF antibody (Kim S. K et al., 2014), or indirectly targeting PAUF by interfering with a key molecule in the PAUF signaling pathway (Cho et al., 2012). Humanized monoclonal antibody may be a desired bio-therapeutic modality with competitiveness such as high affinity and specificity, good stability, and mature engineering and production technology, etc.

Due to the exploratory nature of this research work, several limitations are noted. 1) Although a clear tendency of PAUF expression-dependent cancer metastatic capacity was demonstrated in this study, the correlations were not quantitatively evaluated, likewise, the dose-dependent anti-tumor effects of anti-PAUF antibody were not evaluated either. Although previous *in vitro* studies have suggested dose-dependent effects of PAUF and anti-PAUF treatments on cell signaling activations (Park et al., 2011; Kim et al., 2013) and anti-tumor effects (Kim S. K et al., 2014), respectively, it is necessary to explore dose-response of anti-PAUF against ovarian cancer in future studies. 2) A recent bio-informatic study suggests that the OVCAR-5 cancer cell line may be a metastatic ovarian cancer of upper gastrointestinal origin (Blayney et al., 2016), however, even if our findings may not best represent primary ovarian cancer, they remain highly relevant in ovarian cancer settings, because clinically, up to 20% ovarian cancers are originated from other organs (mainly from the gastrointestinal system) and show poorer prognosis (Chen et al., 2020). Whether cancers metastasized to ovary tend to express higher level of PAUF and thus benefit more from anti-PAUF treatment than primary ovarian cancer are indeed worth of further investigation. 3) In the animal study only one cytotoxic agent, docetaxel, was tested to be combined with anti-PAUF antibody, in the Genomics of Drug Sensitivity in Cancer database (https://www.cancerrxgene.org), docetaxel shows similar cytotoxicity to that of paclitaxel and cisplatin in the OVCAR-5 cell line. In the future studies, combination of anti-PAUF antibody and other chemotherapeutics such as carboplatin and paclitaxel will be tested in different ovarian cancer animal models, e.g., hematogenous spread ovarian cancer models (Coffman et al., 2016) and chemotherapy-resistant models, and tumor metastasis should be evaluated at tissue-level using various methods, such as immunohistochemical assays.

Currently, a non-invasive PAUF detection method is under validation, with which plasma PAUF will be investigated for its diagnostic and prognostic values in pancreatic cancer patients. Meanwhile, as more evidence regarding the immune-suppressive effects of PAUF became available, more studies are ongoing to investigate the anti-PAUF antibody in combination of other immunotherapeutics for treatment of solid tumors including ovarian cancer.

## Conclusion

This study shows that PAUF independently promotes tumor growth and metastasis in ovarian cancer cells, in a baseline PAUF dependent and saturable manner. An anti-PAUF antibody may sensitize and synchronize the anti-tumor effects of cytotoxic agents and prolong survival time for patients of ovarian cancer.

## Data Availability

The original contributions presented in the study are included in the article/Supplementary Material, further inquiries can be directed to the corresponding authors.
