# Supplementary figures and images for: PAUF as a Target for Treatment of High PAUF-Expressing Ovarian Cancer
Source: Front Pharmacol. 2022 May 6;13:890614. doi: 10.3389/fphar.2022.890614 (PMC9121814; doi:10.3389/fphar.2022.890614)

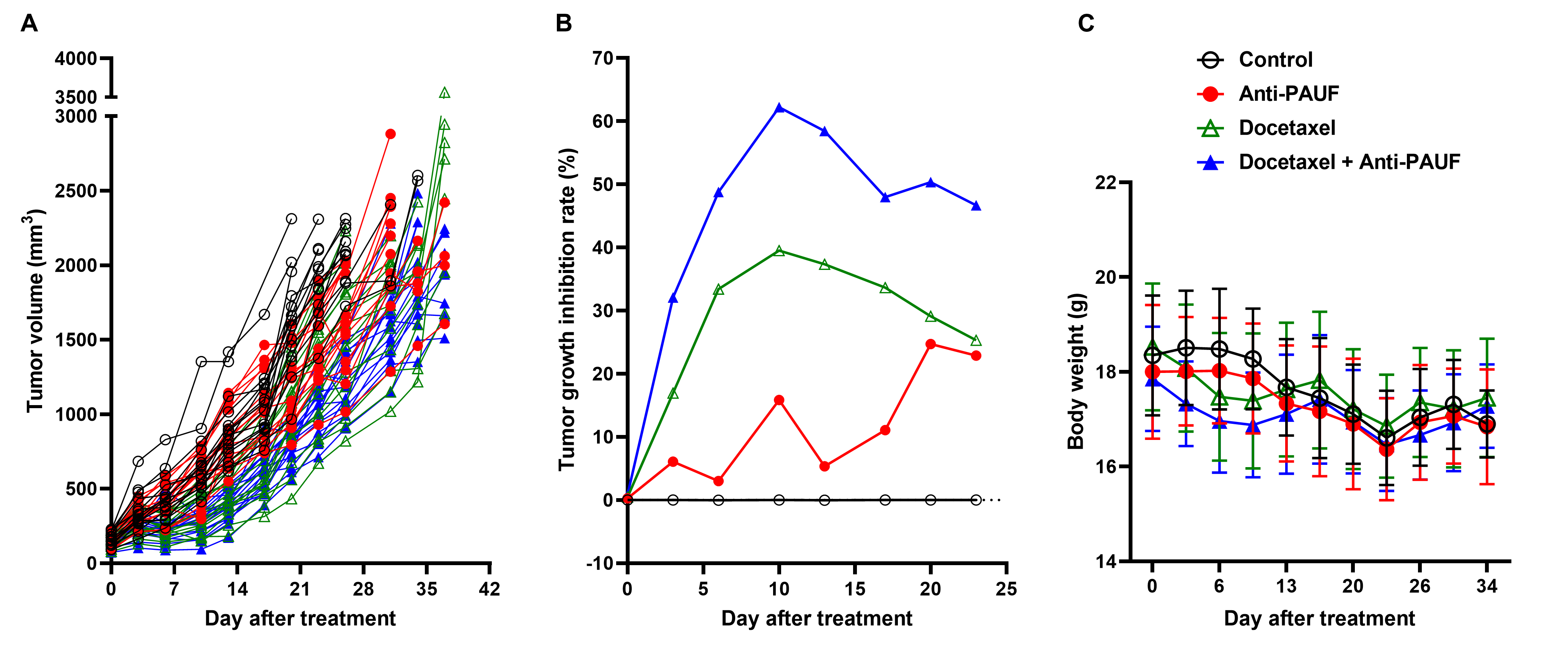

Supplement: Supplementary file 1 [file Image3.TIF]

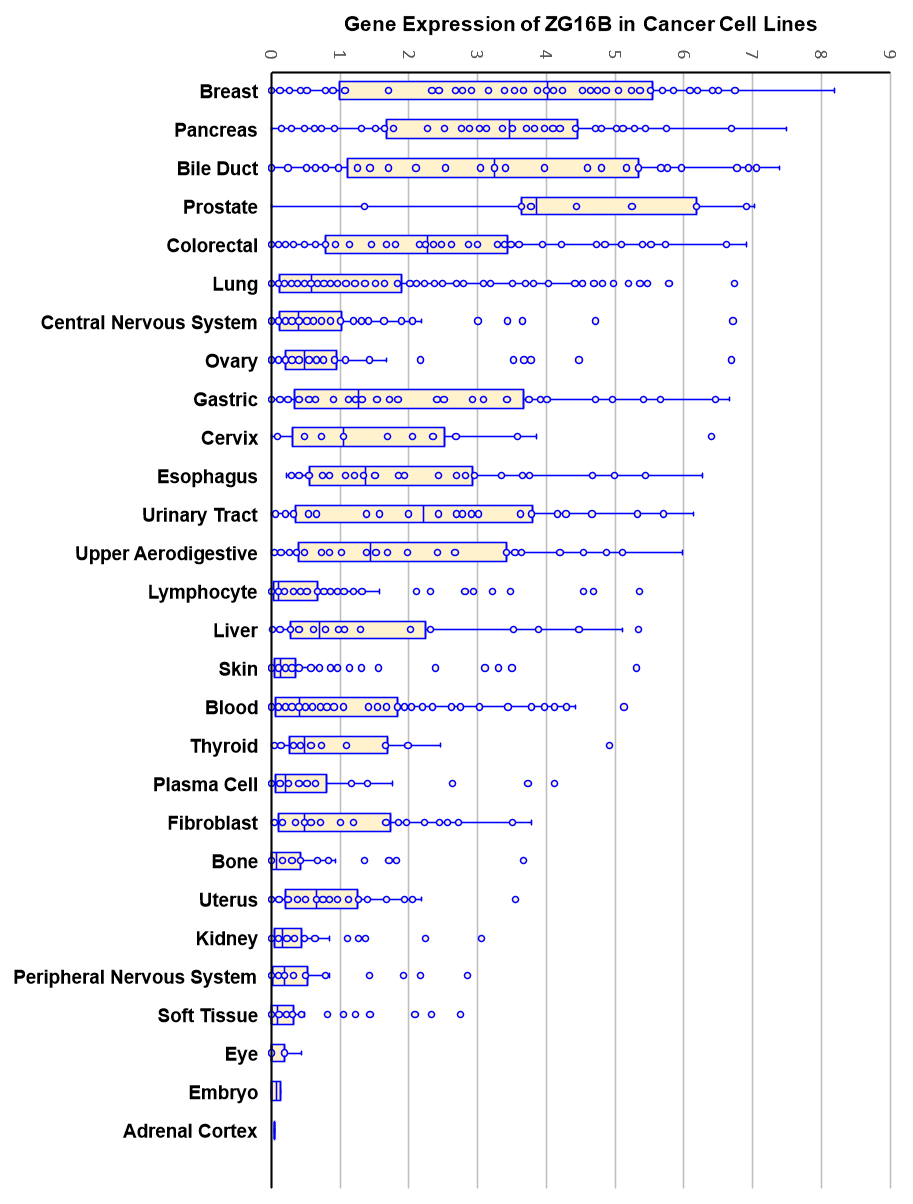

Supplement: Supplementary file 2 [file Image4.TIF]

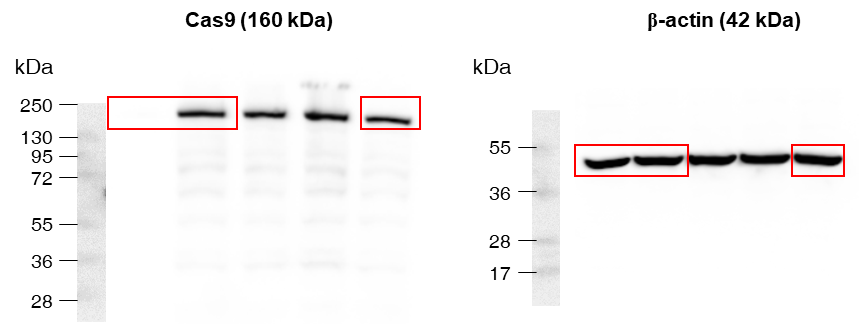

Supplement: Supplementary file 3 [file Image2.TIF]

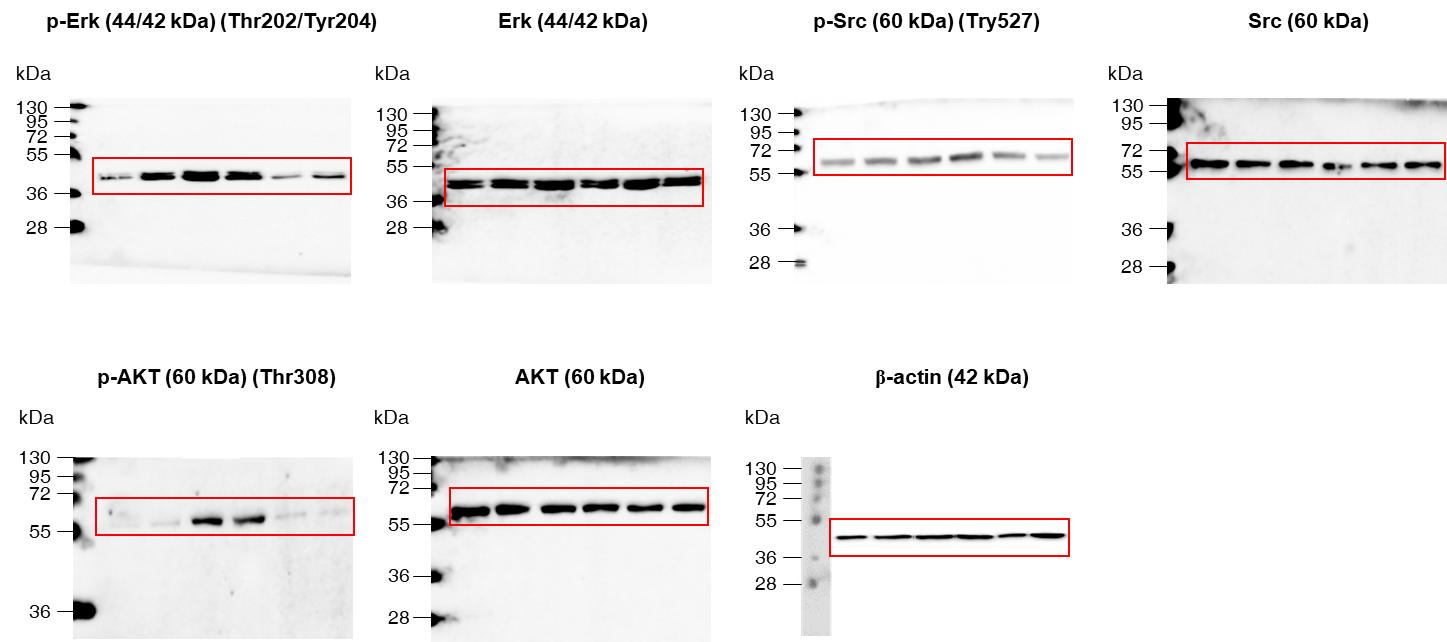

Supplement: Supplementary file 4 [file Image1.TIF]
